# Predictors for bronchoalveolar lavage recovery failure in diffuse parenchymal lung disease

**DOI:** 10.1038/s41598-021-81313-5

**Published:** 2021-01-18

**Authors:** Keigo Koda, Hironao Hozumi, Hideki Yasui, Yuzo Suzuki, Masato Karayama, Kazuki Furuhashi, Noriyuki Enomoto, Tomoyuki Fujisawa, Naoki Inui, Yutaro Nakamura, Takafumi Suda

**Affiliations:** 1 Second Division, Department of Internal Medicine, Hamamatsu University School of Medicine, 1-20-1 Handayama Higashiku, Hamamatsu, 431-3192 Japan; 2 Department of Clinical Pharmacology and Therapeutics, Hamamatsu University School of Medicine, 1-20-1 Handayama Higashiku, Hamamatsu, 431-3192 Japan

**Keywords:** Risk factors, Medical research

## Abstract

Bronchoalveolar lavage (BAL) plays a role in the diagnosis of diffuse parenchymal lung diseases (DPLD); however, poor BAL fluid (BALF) recovery results in low diagnostic reliability. BAL is relatively safe, but its indications should be carefully considered in patients with risks. Therefore, estimating the likelihood of recovery failure is helpful in clinical practice. This study aimed to clarify predictors of BALF recovery failure and to develop its simple-to-use prediction models. We detected the predictors applying a logistic regression model on clinical, physiological, and radiological data from 401 patients with DPLD (derivation cohort). The discrimination performance of the prediction models using these factors was evaluated by the c-index. In the derivation cohort, being a man, the forced expiratory volume in one second/forced vital capacity, and a BAL target site other than right middle lobe or left lingula were independent predictors. The c-indices of models 1 and 2 that we developed were 0.707 and 0.689, respectively. In a separate cohort of 234 patients (validation cohort), the c-indices of the models were 0.689 and 0.670, respectively. In conclusion, we developed and successfully validated simple-to-use prediction models useful for pulmonologists considering BAL indications or target sites, based on independent predictors for BALF recovery failure.

## Introduction

Bronchoalveolar lavage (BAL) is an established bronchoscopic procedure for sampling cellular/humoral components of the lung^1–7^. When combined with adequate clinical information, physical examination, and high-resolution computed tomography (HRCT) images, analysis of retrieved BAL fluid (BALF) may provide strong support for a diagnosis of diffuse parenchymal lung disease (DPLD), including allergic respiratory diseases and interstitial lung diseases (ILD), or it may help narrow the differential diagnosis^1,3–5^. However, the role of BAL in the diagnosis of many DPLDs, except for certain diseases, such as eosinophilic pneumonia, alveolar proteinosis, and alveolar hemorrhage, is not yet well established. Therefore, before performing BAL, it would be necessary to make a list of differential diagnoses and to consider how BAL may be helpful in the diagnosis and management on a case by case basis. Moreover, although BAL is a relatively safe procedure, bronchoscopy with BAL poses some risk of complications including cardiac arrhythmias, hemorrhages, acute exacerbations of asthma, and acute exacerbations of ILD^6,8,9^. Therefore, its indications should be considered carefully based on the patient's tolerance and potential risks. In addition, the BAL procedure’s quality strongly affects the interpretation of BALF results^1^. Poor BALF recovery rates may lower the reliability of BALF results and complicate the differential diagnosis^1,10,11^. Ideally, the BALF recovery rate should be ≥ 30%^1,11^. Therefore, estimating the likelihood of BALF recovery failure prior to performing bronchoscopy may be useful to decide whether or how to perform BAL for undiagnosed patients, especially for those who have some potential risk/contraindications. However, the predictive factors of recovery failure have not been established. We aimed to clarify independent predictors of BALF recovery failure, and to develop a simple-to-use prediction score model that can help define BAL indications using clinical, physiological, and HRCT data in a large cohort of patients with DPLD.

## Methods

### Subjects

**Figure 1:**
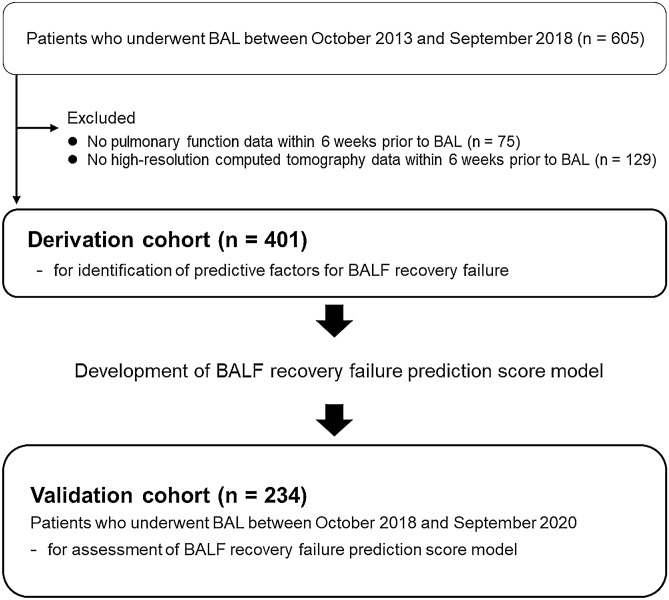
Study flow chart. *BAL* bronchoalveolar lavage, *BALF* BAL fluid.

We retrospectively reviewed records of 605 consecutive patients with DPLD who had undergone elective BAL between October 2013 and September 2018 at the Hamamatsu University Hospital. These patients had been diagnosed as having DPLD on the basis of diagnostic procedure results (including those of BAL) related to guidelines or statements^3–5,12–20^. Figure 1 presents the study flow chart. We excluded data from 75 patients whose pulmonary function test results were unavailable within 6 weeks prior to BAL. Subsequently, we excluded data from 129 patients whose HRCT data were lacking within 6 weeks prior to BAL. Consequently, we analyzed data from 401 patients (derivation cohort) for identification of recovery failure predictors and developed BALF recovery failure prediction score models based on the predictors. Furthermore, to validate the performance of the models, we extracted the data of 234 consecutive patients with DPLD and who had undergone elective BAL between October 2018 and September 2020 at the same hospital (validation cohort). In all patients in this validation cohort, pulmonary function test results and HRCT data within 6 weeks before BAL were available. We conducted the study in accordance with the tenets of the Declaration of Helsinki. The Institutional Review Board of the Hamamatsu University School of Medicine approved this study (approval number 19-306) and waived the need for patient approval or informed consents due to the retrospective nature of the study.

### Data collection

We collected data on the following variables: clinical data, including age, gender, and smoking history (including pack-years); physiological data, including forced vital capacity (FVC), percent predicted FVC (%FVC), forced expiratory volume in one second (FEV_1.0_), and percent predicted FEV_1.0_ (%FEV_1.0_); HRCT data; BAL target site and recovery rate; and diagnosis after BAL (disease category or disease).

### BAL procedure

Well-trained pulmonologists with 8 years or more of experience performed bronchoscopies with BAL, on the basis of the official guidelines^1,7^. Briefly, before the bronchoscopy, the patients inhaled a lidocaine solution through a nebulizer, and got pre-medication consisting of midazolam and pentazocine intravenously administered. The pulmonologists inserted the fiberoptic bronchoscope with 5.9 mm of a distal end outer diameter and 3.0 mm of a channel inner diameter (BF-1TQ290, Olympus, Japan) transorally. During the examination, lidocaine solution was instilled through the instrumentation channel of the bronchoscope for additional local anesthesia. To perform BAL, the pulmonologists placed the tip of the bronchoscope into a wedge position within the selected bronchopulmonary segment (BAL target site), chosen on the basis of an HRCT taken within 6 weeks prior to bronchoscopy. The position of each patient was determined according to BAL target sites; supine position for right middle lobe (RM) or left lingula (LL) targets, left lateral decubitus position for right superior lobe (RS) or right inferior lobe (RI) targets, and right lateral decubitus position for left superior lobe other than LL (LS) or left inferior lobe (LI) targets. A total volume of normal saline of 150 mL (3 aliquots × 50 mL each) were instilled. BALF recovery rates were calculated as the percent (%) rate of the total volume of retrieved BALF to the total instilled volume. BALF recovery failure was defined as a total volume of retrieved BALF lower than 30% of the total instilled volume or aborted BAL due to recovery of less than 5% of each instilled aliquot volume.

### HRCT analysis

Chest HRCT was taken and multi-detector-row CT (MDCT) imaging was performed using a 64-slice MDCT machine (Aquilon-64; Toshiba Medical Systems, Tokyo, Japan) with the patient in the supine position at full inspiration breath hold. Using image analyzing software (SYNAPSE VINCENT; Fuji Film, Tokyo, Japan), we obtained lung volumes and percentages of low attenuation areas (%LAA) in the lung using three-dimensional CT images that were reconstructed from MDCT data. We defined %LAA, a surrogate measurement of emphysema, as the percentage of area below − 950 HU in the total lung area. We used lung volumes and %LAA in the side of BAL target sites for our analyses.

### Statistical analysis

We expressed all values as medians [interquartile ranges (IQRs)] or numbers (%). We applied Fisher’s exact or Chi-squared tests to compare proportions between groups, and the Mann–Whitney U-test to compare medians. We evaluated correlations between different parameters using the Spearman's correlation test. We applied logistic regression analysis to identify variables associated with BAL recovery failure and the calculated odds ratio (OR), 95% confidence interval (CI), and *P* values. We tested all variables identified as significant on the univariate analysis with our multivariate analysis. We performed a receiver-operating characteristic (ROC) curve analysis to identify an optimal cut-off value, chosen as the point with the highest value of sensitivity + specificity − 1. The c-index was calculated as the area under the curve (AUC) in the ROC curve. Using the independent predictors identified, we generated simple point–score models for recovery failure prediction. The discrimination performances of the models were evaluated using the c-index. We considered all *P*-values < 0.05 as indicating statistical significance. In multiple pairwise group comparisons, we performed the Bonferroni correction to adjust the *P*-value. We analyzed all data using EZR (Saitama Medical Center, Jichi Medical University, Saitama, Japan), a graphical user interface for R (The R Foundation for Statistical Computing, Vienna, Austria).

## Results

### Study cohort characteristics

Table 1 summarizes the characteristics of the derivation cohort. The median age at the time of BAL was 69 years, and 64.3% of the patients were men. The median %FVC and FEV_1.0_/FVC were 81.6% and 80.7%, respectively. With respect to HRCT analysis, the median lung volume and %LAA in the side of BAL target site were 1850 mL and 9.8%, respectively. The most common BAL target site was the RM (77.8%), followed by the LL (12.2%). The median BALF recovery rate was 48.7%, and the recovery failure frequency 17.0%.

**Table 1:** Characteristics of derivation cohort.

	n = 401
Age (years)	69 (62–74)
Men/women	258 (64.3)/143 (35.7)
Smoking, never/ex/current	146 (36.4)/196 (48.9)/59 (14.7)
Smoking (pack-years)	18.4 (0–42)
**Pulmonary function**	
FVC (mL)/%FVC (%)	2540 (1920–3060)/81.6 (69.6–94.6)
FEV_1.0_ (mL)/%FEV_1.0_ (%)	1970 (1520–2360)/80.7 (67.7–92.3)
FEV_1.0_/FVC (%)	80.7 (74.5–85.5)
**HRCT analysis**	
Lung volume of BAL side (mL)	1850 (1450–2230)
%LAA in the lung of BAL side (%)	9.8 (4.6–18.1)
**BAL target site**	
Right superior lobe	22 (5.5)
Right middle lobe	312 (77.8)
Right inferior lobe	5 (1.2)
Left superior lobe other than lingula	9 (2.2)
Left lingula	49 (12.2)
Left inferior lobe	4 (1.0)
BALF recovery rate (%)	48.7 (34.7–60.3)
BALF recovery failure, no/ yes	333 (83.0)/68 (17.0)
**Post-BAL diagnosis**	
IIP other than IPF	93 (23.2)
CTD-ILD	70 (17.5)
IPF	44 (11.0)
Sarcoidosis	32 (8.0)
Drug-induced pneumonitis	21 (5.2)
Cryptogenic organizing pneumonia	18 (4.5)
Hypersensitivity pneumonitis	14 (3.5)
Chronic eosinophilic pneumonia	8 (2.0)
Pleuroparenchymal fibroelastosis	7 (1.7)
Others	94 (23.4)

Data are presented as median (IQR), or number (%).

*FVC* forced vital capacity, *%FVC* percent predicted FVC, *FEV*_*1.0*_ forced expiratory volume in one second, *%FEV*_*1.0*_ percent predicted FEV_1.0_, *HRCT* high-resolution computed tomography, *BAL* bronchoalveolar lavage, *BALF* BAL fluid, *LAA* low attenuation area, *IIP* idiopathic interstitial pneumonia, *IPF* idiopathic pulmonary fibrosis, *ILD* interstitial lung disease, *CTD* connective tissue disease.

### BALF recovery and target sites

**Figure 2:**
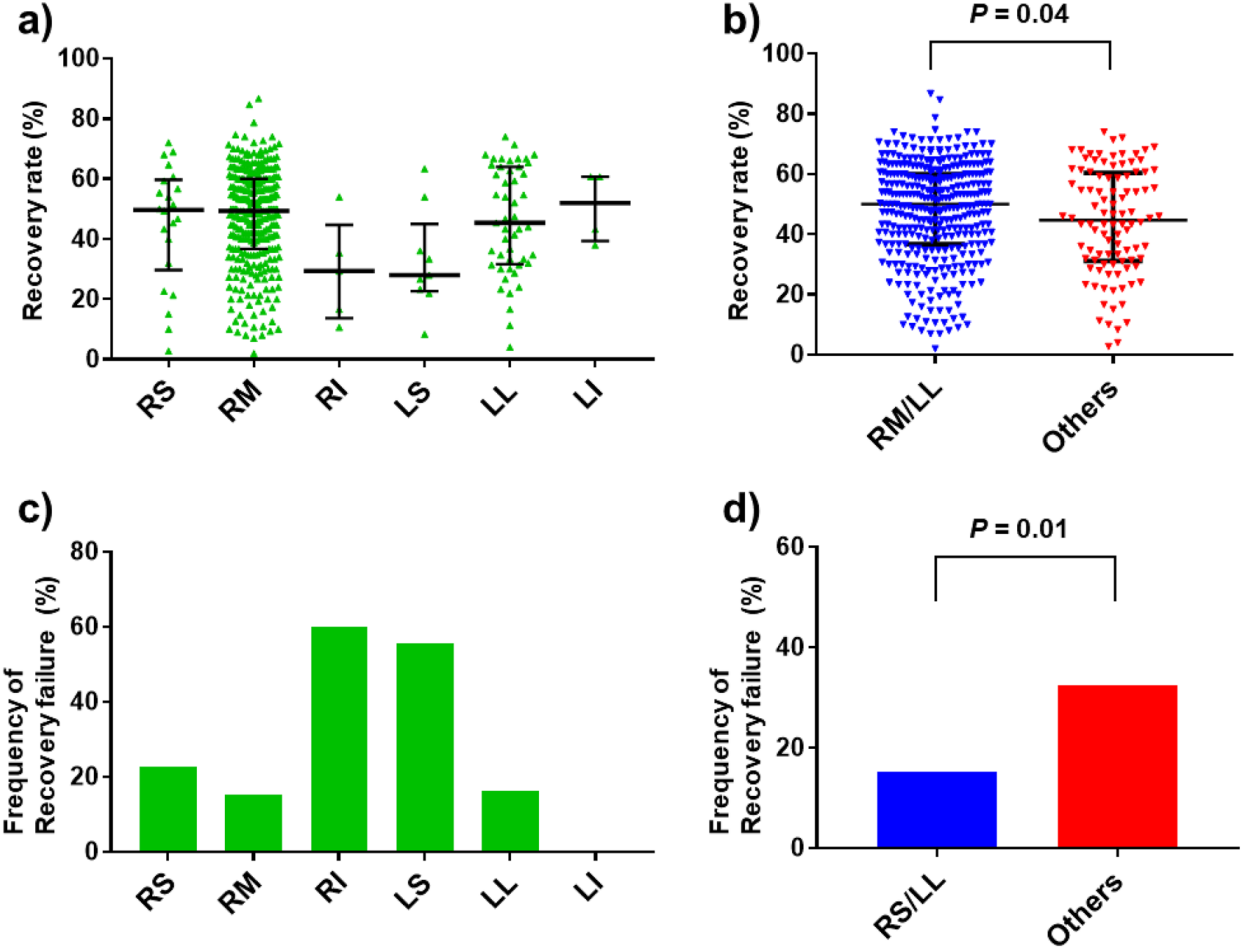
BALF recovery rates and recovery failure frequency in the derivation cohort. (**a**) The median BALF recovery rates (interquartile ranges) from RS, RM, RI, LS, LL, and LI were 49.7% (29.7–59.7), 49.3% (36.7–60.0), 29.7% (13.7–44.7), 28.0% (22.7–45.0), 45.3% (32.7–64.0), and 51.9% (39.3–60.7), respectively. (**b**) The median BALF recovery rate in the RM/LL group was significantly higher than those in the others group (49.3% vs. 43.3%, respectively, *P* = 0.04). (**c**) The BALF recovery failure frequency in RS, RM, RI, LS, LL, and LI were 22.7%, 15.1%, 60.0%, 55.6%, 16.3%, and 0%, respectively. (**d**) The BALF recovery failure frequency in the RM/LL group was significantly lower than those in the others group (15.2% vs. 32.5%, respectively, *P* = 0.01). *BALF* bronchoalveolar lavage fluid, *RS* right superior lobe, *RM* right middle lobe, *RI* right inferior lobe, *LS* left superior lobe other than lingual, *LL* left lingual, *LI* left inferior lobe.

Figure 2a–d show BALF recovery rates and recovery failure frequencies in each target site. We aggregated data from patients who underwent BAL from RM or LL and those who did from a site other than RM or LL (RS, RI, LS, or LI) into an RM/LL group and an others group, respectively; the median recovery rate in the RM/LL group was significantly higher than that in the others group (49.3% vs. 43.3%, respectively, *P* = 0.04) (Fig. 2b). The recovery failure frequency in the RM/LL group was significantly lower than that in the others group (15.2% vs. 32.5%, respectively, *P* = 0.01) (Fig. 2d).

### Clinical, physiological, and radiological parameters and BALF recovery rates

Table 2 presents the correlations of clinical, physiological, and radiological parameters with recovery rates. BALF recovery rates demonstrated a very weak or weak negative correlation with age, smoking (pack-years), and BAL side lung volume, and a weak positive correlation with FEV_1.0_/FVC.

**Table 2:** Correlation of clinical, physiological, and radiological parameters with BALF recovery rate.

	Correlation coefficient	*P*-value
Age (years)	− 0.18	< 0.001*
Smoking (pack-years)	− 0.27	< 0.001*
**Pulmonary function**		
FVC (mL)	− 0.09	0.06
%FVC (%)	0.01	0.90
FEV_1.0_ (mL)	− 0.01	0.81
%FEV_1.0_ (%)	0.10	0.04*
FEV_1.0_/FVC (%)	0.22	< 0.001*
**HRCT analysis**		
Lung volume of BAL side (mL)	− 0.15	< 0.01*
%LAA in BAL side lung (%)	0.01	0.82

*FVC* forced vital capacity, *%FVC* percent predicted FVC, *FEV*_*1.0*_ forced expiratory volume in one second, *%FEV*_*1.0*_ percent predicted FEV_1.0_, *BAL* bronchoalveolar lavage, *BALF* BAL fluid, *LAA* low attenuation area.

*P < 0.05.

### Predictors for BALF recovery failure

Table 3 presents the results of logistic regression analysis for recovery failure. On univariate analyses, being a man (vs. a woman), having high smoking pack-years, high FVC, low FEV_1.0_/FVC, a BAL target site other than RM/LL (vs. RM/LL), and high BAL target site lung volume were associated with recovery failure. On the multivariate analysis, being a man (vs. a woman; OR 5.27, *P* < 0.01), having a low FEV_1.0_/FVC (OR 0.96 per 1% increase, *P* = 0.03), and a BAL target site other than RM/LL (vs. an RM/LL site; OR 2.78, *P* = 0.01) were independent predictors for recovery failure. Table 4 presents the results of logistic regression analysis for recovery failure in the RM/LL group. On univariate analyses, gender (men), high number of smoking pack-years, low FEV_1.0_/FVC, and high BAL target site lung volume were associated with recovery failure. On the multivariate analysis, gender (men vs. women; OR 3.87, *P* < 0.01) and low FEV_1.0_/FVC (OR 0.97 per 1% increase, *P* = 0.04) were independent predictors for recovery failure.

**Table 3:** Results of logistic regression analysis for BALF recovery failure in derivation cohort.

	OR	95% CI	*P*-value
**Univariate**			
Age, per year	1.02	0.99–1.05	0.09
Men (vs. women)	6.02	2.67–13.6	< 0.01*
Smoking, current/ex (vs. never)	4.00	1.98–8.11	< 0.01*
Smoking, per 1 pack-years increase	1.02	1.01–1.03	< 0.01*
**Pulmonary function**			
FVC, per 100 mL increase	1.03	1.001–1.07	0.04*
%FVC, per 1% increase	1.00	0.99–1.02	0.81
FEV_1.0_, per 100 mL increase	1.02	0.98–1.06	0.37
%FEV_1.0_, per 1% increase	0.99	0.98–1.01	0.30
FEV_1.0_/FVC, per 1% increase	0.96	0.94–0.99	< 0.01*
**BAL target site**			
Right (vs. left)	0.59	0.32–1.08	0.09
Superior lobe^¶^ (vs. inferior lobe)	0.95	0.20–4.61	0.95
Inferior lobe (vs. RM/LL)	2.78	0.68–11.1	0.16
Superior lobe^¶^ (vs. RM/LL)	2.63	1.18–5.88	0.02*
Other than RM/LL (vs. RM/LL)	2.63	1.28–5.56	< 0.01*
**HRCT analysis**			
Lung volume of BAL side, 100 mL increase	1.06	1.02–1.11	< 0.01*
%LAA in BAL side, per 1% increase	1.00	0.97–1.02	0.86
**Multivariate**			
Men (vs. women)	5.27	2.00–13.9	< 0.01*
Smoking, per 1 pack-years increase	1.01	0.99–1.02	0.21
FVC, per 100 mL increase	0.96	0.91–1.01	0.14
FEV_1.0_/FVC, per 1% increase	0.97	0.94–0.99	0.03*
BAL target site, other than RM/LL (vs. RM/LL)	2.78	1.27–5.88	0.01*
Lung volume of BAL side, 100 mL increase	1.05	0.98–1.12	0.18

*BAL* bronchoalveolar lavage, *BALF* BAL fluid, *OR* odds ratio, *CI* confidence interval, *FVC* forced vital capacity, *%FVC* percent predicted FVC, *FEV*_*1.0*_ forced expiratory volume in one second, *%FEV*_*1.0*_ percent predicted FEV_1.0_, *RM/LL* right middle lobe or left lingual, *LAA* low attenuation area.

*P < 0.05.

^¶^Other than lingula.

**Table 4:** Logistic regression analysis for BALF recovery failure in the RM/LL group.

	OR	95% CI	*P*-value
**Univariate**			
Age, per year	1.03	0.99–1.05	0.09
Men (vs. women)	5.64	2.34–13.6	< 0.01*
Smoking, current/ex (vs. never)	3.95	1.80–8.65	< 0.01*
Smoking, per 1 pack-years increase	1.02	1.01–1.03	< 0.01*
**Pulmonary function**			
FVC, per 100 mL increase	1.03	0.99–1.07	0.08
%FVC, per 1% increase	1.00	0.98–1.02	0.99
FEV_1.0_, per 100 mL increase	1.02	0.97–1.06	0.50
%FEV_1.0_, per 1% increase	0.99	0.97–1.01	0.19
FEV_1.0_/FVC, per 1% increase	0.96	0.93–0.99	< 0.01*
BAL target site, ML (vs. LL)	0.91	0.40–2.06	0.82
**HRCT analysis**			
Lung volume of BAL side, 100 mL increase	1.08	1.03–1.13	< 0.01*
%LAA in BAL side, per 1% increase	0.99	0.96–1.02	0.70
**Multivariate**			
Men (vs. women)	3.87	1.44–10.4	< 0.01*
Smoking, per 1 pack-years increase	1.01	0.99–1.02	0.27
FEV_1.0_/FVC, per 1% increase	0.97	0.94–0.99	0.04*
Lung volume of BAL side, 100 mL increase	1.00	0.99–1.01	0.42

*BAL* bronchoalveolar lavage, *BALF* BAL fluid, *OR* odds ratio, *CI* confidence interval, *FVC* forced vital capacity, *%FVC* percent predicted FVC, *FEV*_*1.0*_ forced expiratory volume in one second, *%FEV*_*1.0*_ percent predicted FEV_1.0_, *RM* right middle lobe, *LL* left lingual, *LAA* low attenuation area.

*P < 0.05.

The Supplementary Table S1 presents the comparison of diagnoses after BAL between men and women in the derivation cohort. The proportion of patients with idiopathic pulmonary fibrosis (IPF) was significantly higher in men than in women. Meanwhile, the proportions of those with connective tissue disease-associated ILD (CTD-ILD) and those with sarcoidosis were significantly lower in men than in women. The Supplementary Table S2 presents the results of the logistic regression analysis for recovery failure with adjustment for diagnosis after BAL. These disease-adjusted multivariate analyses also identified being a man (vs. a woman), and having low FEV_1.0_/FVC, and a BAL target site other than RM/LL as independent predictors for recovery failure, irrespective of background disease/disease categories.

We identified the optimal cut-off value of FEV_1.0_/FVC for predicting recovery failure in the derivation cohort using a ROC curve analysis (Supplementary Fig. S1). The c-index was 0.60 (95% CI 0.520–0.676). Using 74.4% as the cut-off value of FEV_1.0_/FVC, the sensitivity and specificity were 80.0% and 36.9%, respectively.

### Development of the BALF recovery failure prediction score model

**Figure 3:**
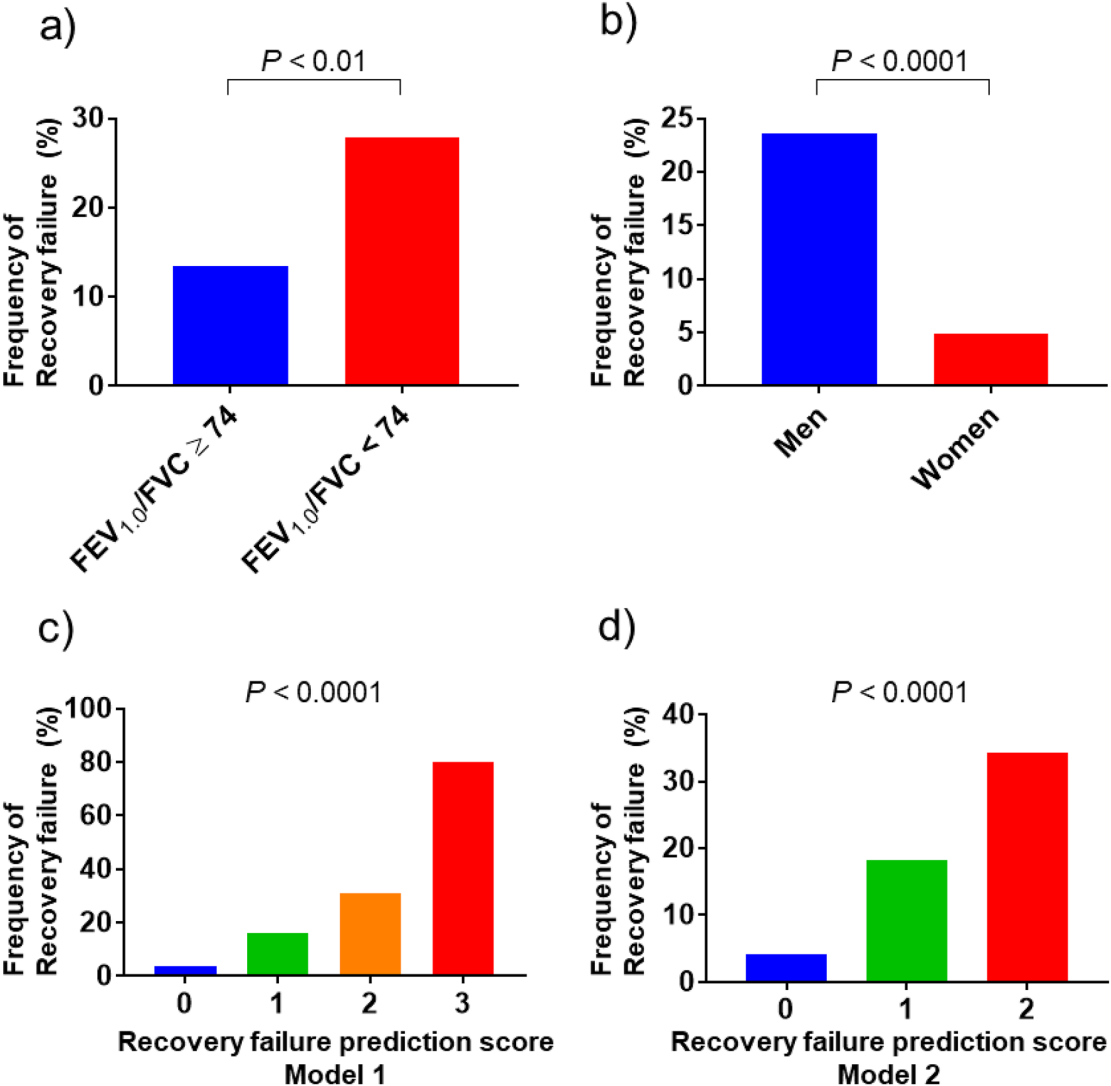
Predictive factors and recovery failure frequency in the derivation cohort. (**a**) The recovery failure frequency in patients who showed an FEV_1.0_/FVC < 74% was significantly higher than that in those with FEV_1.0_/FVC ≥ 74% (27.8% vs. 13.5%, respectively; P < 0.01). (**b**) The recovery failure frequency in men was significantly higher than that in women (23.6% vs. 4.9%, respectively; P < 0.0001). (**c**) In model 1, each predictor (being a man, FEV_1.0_/FVC < 74%, a BAL target site other than the RM/ LL) was assigned one point. The recovery failure frequencies in the model 1 prediction score groups (total scores 0, 1, 2, and 3) were 3.6%, 16.2%, 30.9%, and 80.0%, respectively (*P* < 0.0001; c-index 0.70). (**d**) In model 2, each predictor (being a man and FEV_1.0_/FVC < 74%) was assigned one point. The recovery failure frequencies in the model 2 prediction score groups (total scores 0, 1, and 2) were 4.2%, 18.2%, and 34.3%, respectively (*P* < 0.0001; c-index 0.69). FEV_1.0_, forced expiratory volume in one second, FVC forced vital capacity.

Using the independent predictors identified, including gender, FEV_1.0_/FVC, and BAL target site, we attempted to develop a simple point-score model for recovery failure prediction. We determined an FEV_1.0_/FVC cut-off value at 74% based on the result of the ROC analysis that was performed earlier in the derivation cohort. In this cohort, the recovery failure frequency in patients who showed an FEV_1.0_/FVC < 74% was significantly higher than that in those who with an FEV_1.0_/FVC ≥ 74% (27.8% vs. 13.5%; *P* < 0.01; Fig. 3a). The recovery failure frequency in men was significantly higher than that in women (23.6% vs. 4.9%; *P* < 0.0001; Fig. 3b). The recovery failure frequency in patients who underwent BAL in a target site other than RM/LL was significantly higher than that in those who had an RM/LL target site (32.5% vs. 15.2%; *P* < 0.01). We assigned 1 point to each predictor, and categorized patients of the derivation cohort into four groups based on their total point scores (0–3) (model 1). Figure 3c presents the model 1 performance. The recovery failure frequencies for the prediction score groups (0, 1, 2, and 3) were 3.6%, 16.2%, 30.9%, and 80.0%, respectively (*P* < 0.0001). The c-index of this model was 0.707 (95% CI 0.648–0.766) (Supplementary Fig. S2a). In a similar manner, we assigned 1 point for having low FEV_1.0_/FVC and for being a man, and we categorized the patients of the derivation cohort into three groups based on their total point scores (0–2) (model 2, Fig. 3d). The recovery failure frequencies in the recovery failure prediction score groups (0, 1, and 2) were 4.2%, 18.2%, and 34.3%, respectively (*P* < 0.0001). The c-index of this model was 0.689 (95% CI 0.631–0.746) (Supplementary Fig. S2b).

### Validation of the BALF recovery failure prediction score model

**Figure 4:**
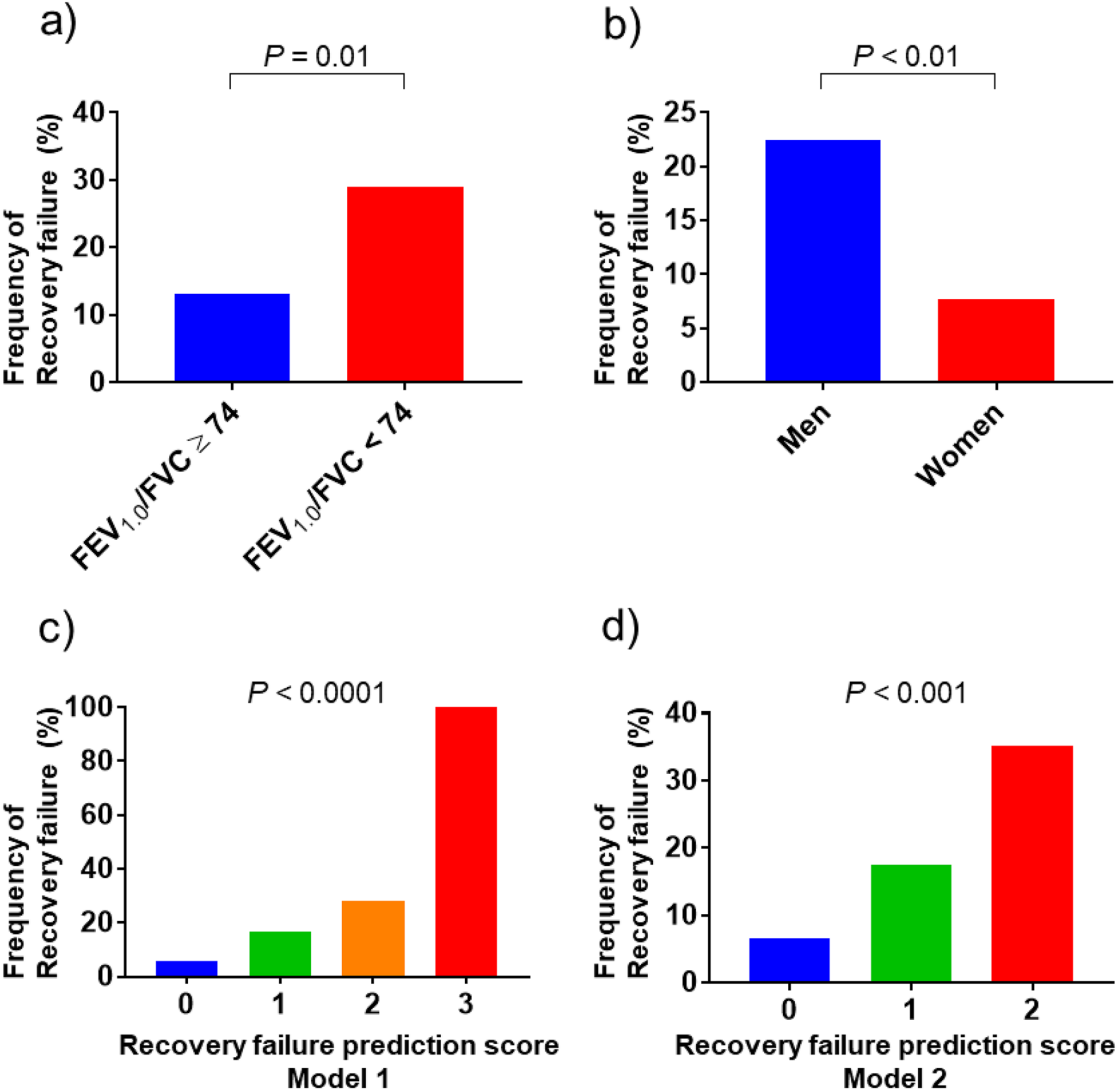
Predictive factors and recovery failure frequency in the validation cohort. (**a**) The recovery failure frequency is significantly higher in patients with an FEV_1.0_/FVC < 74% than in those with an FEV_1.0_/FVC ≥ 74% (28.9% vs. 13.2%, *P* = 0.01). (**b**) The recovery failure frequency is significantly higher in men than in women (22.4% vs. 7.7%, *P* < 0.01). (**c**) In model 1, each predictor (i.e., being a man, FEV_1.0_/FVC < 74%, BAL target site other than the RM/ LL) was assigned one point. The recovery failure frequencies in the prediction score groups (total scores 0, 1, 2, and 3) are 5.6%, 16.5%, 28.0%, and 100%, respectively (*P* < 0.0001; c-index 0.69). (d) In model 2, each predictor (i.e., being a man and FEV_1.0_/FVC < 74%) was assigned one point. The recovery failure frequencies in the prediction score groups (total scores 0, 1, and 2) are 6.6%, 17.4%, and 35.1%, respectively (*P* < 0.001; c-index 0.67). FEV_1.0_, forced expiratory volume in one second; FVC, forced vital capacity.

The characteristics of the validation cohort are summarized in Supplementary Table S3. In this cohort, the recovery failure frequency was significantly higher in patients with an FEV_1.0_/FVC < 74% than in those with an FEV_1.0_/FVC ≥ 74% (28.9% vs. 13.2%, *P* = 0.01, Fig. 4a); in men than in women (22.4% vs. 7.7%, *P* < 0.01, Fig. 4b); and in patients who underwent BAL on a target site other than the RM/LL than in those who had an RM/ LL target site (34.8% vs. 14.8%, *P* = 0.02). Figure 4c presents the performance of model 1. The recovery failure frequencies for the prediction score groups 0, 1, 2, and 3 were 5.6%, 16.5%, 28.0%, and 100.0%, respectively (*P* < 0.0001). The c-index of this model was 0.689 (95% CI 0.606–0.772) (Supplementary Fig. S2c). Figure 4d presents the performance of model 2. The recovery failure frequencies in the recovery failure prediction score groups 0, 1, and 2 were 6.6%, 17.4%, and 35.1%, respectively (*P* < 0.001). The c-index of this model was 0.670 (95% CI 0.588–0.753) (Supplementary Fig. S2d).

## Discussion

Our multivariate logistic regression analysis revealed that being a man (vs. a woman), having low FEV_1.0_/FVC, and a BAL target site other than RM/LL (vs. RM/LL) were independently associated with a higher frequency of BALF recovery failure. Using these independent predictive factors, we built BALF recovery failure prediction score models that are simple to use for risk determination. We successfully validated our prediction score models, based on the comparable discrimination between two separate cohorts. To our knowledge, this is the first and largest study identifying independent predictors of BALF recovery failure based on clinical, physiological, and radiological data in patients with DPLD, and the first study to propose simple-to-use recovery failure prediction score models.

Retrospective studies on BAL recovery exist, Schildge et al. and Karimi et al. demonstrated that BALF recovery rates were weakly correlated with age, smoking history (pack-years), and FEV_1.0_/FVC, although these were based on only bivariate analyses^10,21^. A reduced compliance in the lung parenchyma caused by aging or smoking may easily induce a collapse of the airway during BAL^21^. Our results are consistent with those findings. Furthermore, we identified the independent predictors of recovery failure based on multivariate logistic analyses in a large cohort of patients with DPLD, which is an advantage of this study.

We also found that being a man (vs. a woman) was an independent predictor of recovery failure, regardless of adjustments for smoking history, pulmonary function test results, BAL target site, and lung volume. Although the proportions of patients with CTD-ILD, those with IPF, and those with sarcoidosis were different between men and women in this study, our multivariate analyses adjusted for post-BAL diagnosis also demonstrated that being a man was an independent predictor. Therefore, it is unlikely that the background disease composition difference between men and women affected our results. Li et al. demonstrated that women have a significantly smaller bronchial lumen diameter and cross-sectional lumen area than men, irrespective of smoking status^22^. Gender differences in anatomy of the lower airway (e.g., the difference in the cross-sectional area or volume of peripheral structures beyond a wedged bronchus) may affect BAL recovery.

A guideline on BAL recommended a BALF recovery rate of ≥ 30% to obtain an optimal alveolar sample and for safety reasons and that BAL be discontinued if the recovery volume is too low^1^. In patients with ILD and had a BALF recovery rate of ≥ 30%, Schildge et al. found no significant difference in the cell count between the higher and lower recovery rate groups^10^. This suggested that in patients with ILD, the BALF recovery rate may not have a significant impact on diagnosis if the rate is ≥ 30%. On the other hand, except for studies on infectious diseases, there is insufficient evidence on whether a BALF recovery rate of < 30% reflects the true cell count from the distal airspaces or whether it can contribute to the diagnosis of DPLD^23^. The required BALF recovery rate cutoff value for diagnosis likely varies depending on the disease. Because the present study aimed to identify the predictors of BALF recovery failure, we did not assess whether low recovery rate or failure affected cell count or diagnosis. Further studies are needed to clarify this issue.

In addition, the guideline suggested that the target site should be selected based on the HRCT rather than selecting the RM/LL^1^. However, the optimal target site varies among cases^24–26^, and evidence on this has not been fully established. In cases that have HRCT abnormalities at various sites, including the RM/LL, attending physicians may be unsure on the selection of a target site between sites other than the RM/LL with the most prominent abnormalities and the RM/ LL with some extent of abnormality, the latter being the traditional sites with high BAL recovery rate. In this context, by determining the risk of BAL recovery failure, our simple to use score model may serve as a guide when choosing the target site on which to perform BAL. For instance, if a man with DPLD has low FEV_1.0_/FVC, a BAL target site other than RM/LL should probably be avoided to minimize the likelihood of recovery failure. On the other hand, when there are no abnormalities in RM/LL, selection of other target sites with prominent HRCT abnormalities should be considered. However, if such cases are suspected to be at risk for potential complications or have contraindications, BAL recovery failure may only do harm not give benefit. Therefore, determining the risk of BAL recovery failure, in addition to the diagnostic yield and impact on patient management of BAL, may help determine whether BAL or an alternative test is needed. Collectively, these models can provide helpful information to select a BAL target site or to consider BAL indications for patients with risks.

In this study, we determined 74% as the FEV_1.0_/FVC cut-off value for recovery failure in our prediction model on the basis of the result of the ROC analysis regardless of the standard spirometric criterion for airflow limitation being at FEV_1.0_/FVC < 70%^27^. We also evaluated the performance of our prediction model using a cut-off value at 70%; however, the performance was comparable to that at 74%. A larger study is needed to determine the optimal FEV_1.0_/FVC cut-off value.

We are aware of the limitations in our study. First, the retrospective design of the study renders it vulnerable to several biases. For instance, because our institution is a regional referral center, selection bias in our study population is a possibility. Second, this study included patients with a variety of DPLDs. Therefore, the physiological and/or morphological differences among diseases may have affected BALF recovery rates. Third, the BALF recovery rate may have been affected by factors other than those examined in the present study; these include suction pressure, individual anatomical differences, and the diameter of the bronchial segments. Finally, BAL guidelines recommend that the total volume of normal saline instilled should be between 100 and 300 mL divided into 3 to 5 aliquots^1,7^, which yields some variability in the real-world BAL protocols. In our study, we consistently used 150 mL (3 aliquots of 50 mL each) for the BAL protocol. Differences in instilled volume may be related to both the recovery rate and safety of BAL. The optimal volume to be instilled would need to be established, especially in patients at risk, including those with hypoxemia. A different study should analyze associations between the total instilled volume/aliquots, recovery failure, and safety.

In conclusion, our results revealed that being a man, having a low FEV_1.0_/FVC, and a BAL target site other than RM/LL were independent predictors of BALF recovery failure in patients with DPLD, and they suggest that simple-to-use score models based on these predictors are helpful for predicting recovery failure. Our results will provide valuable information for pulmonologists choosing a BAL target site and weighing the potential benefits against the burdens of BAL procedures. A prospective, multicentre study is required to validate these results.

## Supplementary Information

Supplementary Information

## Data Availability

The data that support the findings of this study are available from the Hamamatsu University School of Medicine but restrictions apply to the availability of these data, which were used under license for the current study, and so are not publicly available. Data are however available from the authors upon reasonable request and with permission of the Hamamatsu University School of Medicine.
