# Supplementary material for: Predictors for bronchoalveolar lavage recovery failure in diffuse parenchymal lung disease
Source: Sci Rep. 2021 Jan 18;11:1682. doi: 10.1038/s41598-021-81313-5 (PMC7814131; doi:10.1038/s41598-021-81313-5)
Supplement: Supplementary file 1 — Supplementary Information [file 41598_2021_81313_MOESM1_ESM.docx]

**Supplementary Information**

**Title: Predictors for bronchoalveolar lavage recovery failure in diffuse parenchymal lung disease**

**Authors’ full names:**

Keigo Koda, MD^1 ‡^, Hironao Hozumi, MD, PhD^1 ‡ *^, Hideki Yasui, MD, PhD^1^, Yuzo Suzuki, MD, PhD^1^, Masato Karayama, MD, PhD^1^, Kazuki Furuhashi, MD, PhD^1^, Noriyuki Enomoto, MD, PhD^1^, Tomoyuki Fujisawa, MD, PhD^1^, Naoki Inui, MD, PhD^1, 2^, Yutaro Nakamura, MD, Ph.D.^1^, Takafumi Suda, MD, PhD^1^

^‡^ Dr Keigo Koda and Dr Hironao Hozumi contributed equally to this paper

**Authors’ affiliations:**

^1^ Second Division, Department of Internal Medicine, Hamamatsu University School of Medicine, Hamamatsu, 1-20-1 Handayama Higashiku, Hamamatsu 431-3192, Japan

^2^ Department of Clinical Pharmacology and Therapeutics, Hamamatsu University School of Medicine, Hamamatsu, 1-20-1 Handayama Higashiku, Hamamatsu 431-3192, Japan

**Supplementary Table S1. Post-BAL diagnosis and gender**

| Disease category | **Men, n = 258** | **Women, n = 143** | ***P*-value** |
| --- | --- | --- | --- |
| IIP other than IPF | 62 (24.0) | 31 (21.7) | 0.62 |
| Connective tissue disease-associated ILD | 25 (9.7) | 45 (31.5) | <0.01* |
| IPF | 41 (15.9) | 3 (2.1) | <0.01* |
| Sarcoidosis | 14 (5.4) | 18 (12.6) | 0.02* |
| Drug-induced pneumonitis | 17 (6.6) | 4 (2.8) | 0.16 |
| Cryptogenic organizing pneumonia | 14 (5.4) | 4 (2.8) | 0.32 |
| Hypersensitivity pneumonitis | 12 (4.7) | 2 (1.4) | 0.15 |
| Chronic eosinophilic pneumonia | 5 (1.9) | 3 (2.1) | 0.99 |
| Pleuroparenchymal fibroelastosis | 5 (1.9) | 2 (1.4) | 0.99 |
| Others | 63 (24.4) | 31 (21.7) | 0.62 |

Data are presented as number (%).

**P* < 0.05

BAL, bronchoalveolar lavage; IIP, idiopathic interstitial pneumonia; IPF, idiopathic pulmonary fibrosis; ILD, interstitial lung disease.

**Supplementary Table S2. Results of logistic regression analysis for BALF recovery failure in derivation cohort; disease adjustment**

| **Multivariate** |  | **Model 1** |  |  | **Model 2** |  |  | **Model 3** |  |  | **Model 4** |  |
| --- | --- | --- | --- | --- | --- | --- | --- | --- | --- | --- | --- | --- |
|  | **OR** | **95%** **CI** | ***P*-value** | **OR** | **95%** **CI** | ***P*-value** | **OR** | **95%** **CI** | ***P*-value** | **OR** | **95%** **CI** | ***P*-value** |
| Men (vs. women) | 4.94 | 1.86–13.1 | <0.01* | 5.25 | 1.95–14.1 | <0.01* | 5.18 | 1.95–13.8 | <0.01* | 5.08 | 1.92–13.5 | <0.01* |
| Smoking,  per 1 pack-years increase | 1.01 | 0.99–1.02 | 0.19 | 1.01 | 0.99–1.02 | 0.22 | 1.01 | 0.99–1.02 | 0.23 | 1.01 | 0.99–1.02 | 0.23 |
| FVC,  per 100mL increase | 0.96 | 0.91–1.02 | 0.15 | 0.96 | 0.91–1.01 | 0.14 | 0.96 | 0.91–1.01 | 0.14 | 0.96 | 0.91–1.01 | 0.15 |
| FEV_1.0_/FVC,  per 1% increase | 0.97 | 0.94–0.99 | 0.02* | 0.97 | 0.94–0.99 | 0.03* | 0.97 | 0.94–0.99 | 0.03* | 0.97 | 0.94–0.99 | 0.03* |
| BAL target site, other than  RM/LL (vs. RM/LL) | 2.78 | 1.28–5.88 | 0.01* | 2.78 | 1.27–5.88 | 0.01* | 2.78 | 1.28–6.25 | 0.01* | 2.70 | 1.25–5.88 | 0.01* |
| Lung volume of BAL side,  100mL increase | 1.05 | 0.98–1.12 | 0.17 | 1.04 | 0.98–1.12 | 0.18 | 1.04 | 0.98–1.12 | 0.18 | 1.05 | 0.98–1.12 | 0.17 |
| Post-BAL diagnosis |  |  |  |  |  |  |  |  |  |  |  |  |
| IIP other than IPF | 1.32 | 0.72–2.41 | 0.38 |  |  |  |  |  |  |  |  |  |
| CTD-ILD |  |  |  | 0.98 | 0.41–2.34 | 0.97 |  |  |  |  |  |  |
| IPF |  |  |  |  |  |  | 1.14 | 0.49–2.63 | 0.76 |  |  |  |
| Sarcoidosis |  |  |  |  |  |  |  |  |  | 0.67 | 0.18–2.48 | 0.55 |

**P* < 0.05

BAL, bronchoalveolar lavage; BALF, BAL fluid; OR, odds ratio; CI, confidence interval; FVC, forced vital capacity; %FVC, percent predicted FVC; FEV_1.0_, forced expiratory volume in one second; %FEV_1.0_, percent predicted FEV_1.0_; RM/LL, right middle lobe or left lingula; LAA, low attenuation area. IIP, idiopathic interstitial pneumonia; IPF, idiopathic pulmonary fibrosis; CTD, connective tissue disease; ILD, interstitial lung disease.

**Supplementary Table S3. Characteristics of the validation cohort**

|  | n = 234 |
| --- | --- |
| Age (years) | 66 (57–72) |
| Men/women | 143 (61.1)/ 91 (38.9) |
| Smoking, never/ex/current/unknown | 98 (41.9)/ 96 (41.0)/ 24 (10.3)/ 16 (6.8) |
| Smoking (pack years) | 11 (0–35) |
| Pulmonary function |  |
| FVC (mL) | 2530 (1990–3200) |
| %FVC (%) | 82.5 (69.5–96.8) |
| FEV_1.0_ (mL) | 2070 (1630–2490) |
| %FEV_1.0_ (%) | 84.6 (70.3–92.7) |
| FEV_1.0_/ FVC (%) | 81.1 (76.1–86.3) |
| BAL target site |  |
| Right upper lobe | 11 (4.7) |
| Right middle lobe | 170 (72.7) |
| Right lower lobe | 7 (3.0) |
| Left upper lobe other than the lingula | 3 (1.3) |
| Lingula | 41 (17.5) |
| Left lower lobe | 2 (0.9) |
| BALF recovery failure, yes | 39 (16.7) |
| BALF recovery failure prediction score |  |
| Model 1, 0/1/2/3 | 72 (30.8)/ 109 (46.6)/ 50 (21.4)/ 3 (1.3) |
| Model 2, 0/1/2 | 76 (32.5)/ 121 (51.7)/ 37 (15.8) |

Data are presented as median (IQR) or number (%)

FVC, forced vital capacity; %FVC, percent predicted FVC; FEV_1.0_, forced expiratory volume in one second; %FEV_1.0_, percent predicted FEV_1.0_; BAL, bronchoalveolar lavage; BALF, BAL fluid**Supplementary Figure S1.** Receiver operating characteristic curve


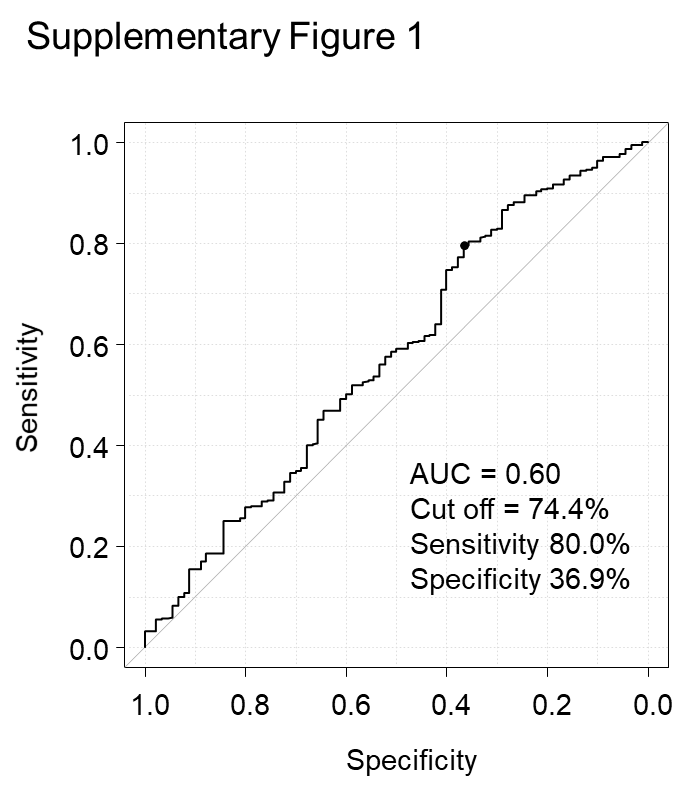


The receiver operating characteristic analysis was performed to identify the optimal cut-off value of FEV_1.0_/FVC for predicting BALF recovery failure in the derivation cohort. The area under the curve (c-index) was 0.60 (95% CI 0.52–0.676). Using 74.4% as the cut-off value of FEV_1.0_/FVC, the sensitivity and specificity were 80.0% and 36.9%, respectively.**Supplementary Figure S2.** Receiver-operating characteristic curves of the prediction score models of BALF recovery failure


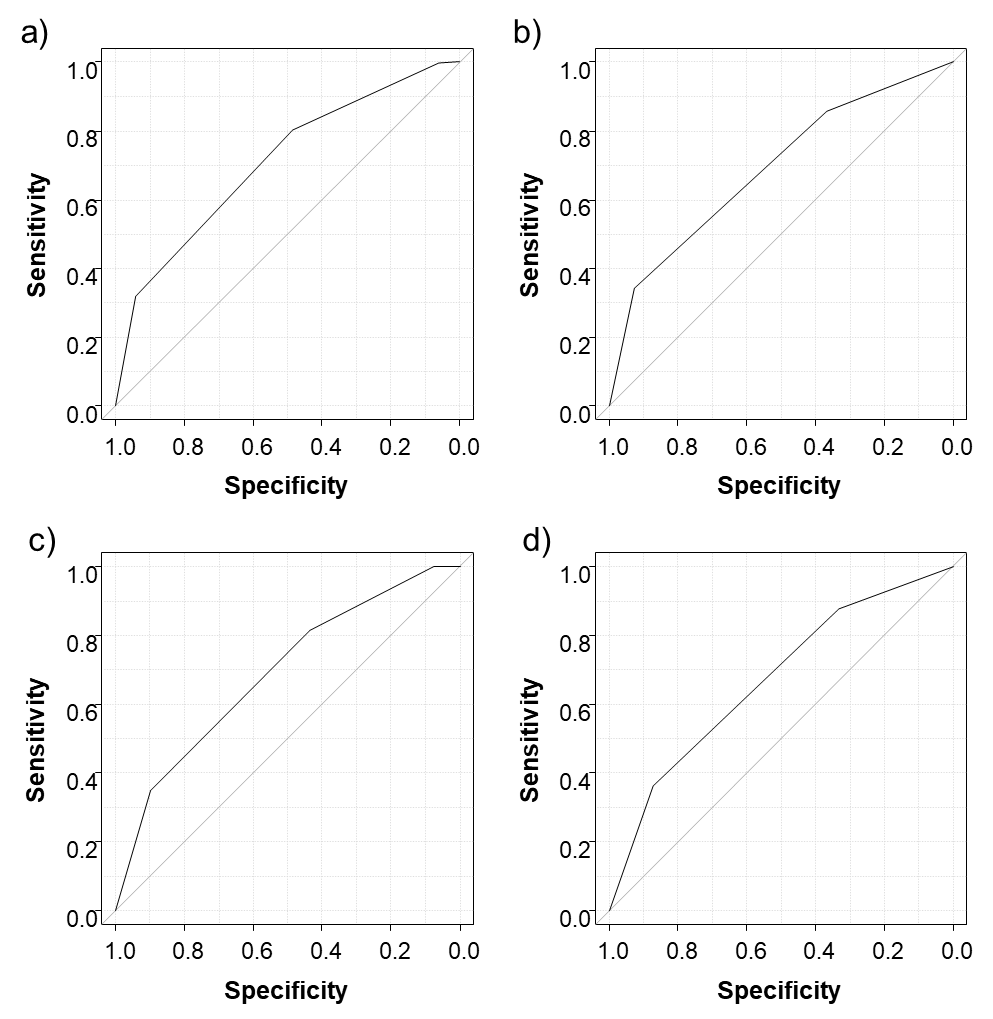


1. Model 1 in the derivation cohort
2. Model 2 in the derivation cohort
3. Model 1 in the validation cohort
4. Model 2 in the validation cohort
